# TRIM50 suppressed hepatocarcinoma progression through directly targeting SNAIL for ubiquitous degradation

**DOI:** 10.1038/s41419-018-0644-4

**Published:** 2018-05-22

**Authors:** Xiaoxiao Ma, Xiaomin Ma, Yumin Qiu, Lihui Zhu, Yueke Lin, Yajing You, Dapeng Ma, Zhenzhi Qin, Caiyu Sun, Yunxue Zhao, Yanlin Sun, Lihui Han

**Affiliations:** 1 Department of Immunology, Shandong Provincial Key Laboratory of Infection & Immunology, Shandong University School of Basic Medical Sciences, 250012 Jinan, China; 2 Department of Pharmacology, Shandong University School of Basic Medical Sciences, 250012 Jinan, China; 3 Department of Pathology, Shandong University School of Basic Medical Sciences, 250012 Jinan, China

## Abstract

Tripartite motif-containing 50 (TRIM50) belongs to the tripartite motif (TRIM) protein family, which has been implicated in the pathogenesis of multiple cancers. However, the role of TRIM50 in hepatocellular carcinoma (HCC) remains to be clarified. Here we showed that TRIM50 expression was significantly decreased in liver cancer tissues compared with corresponding non-cancerous liver tissues, and its decreased expression was significantly correlated with advanced disease progression. Gain-of-function assay by exogenous overexpression of TRIM50 in HCC cells showed that proliferation, colony formation, migration and invasion of HCC cells were significantly inhibited, whereas loss-of-function assay by TRIM50 knockdown showed that these malignant behaviors of HCC cells were significantly increased. Further investigation showed that TRIM50 could directly bind with SNAIL and induced K-48 linked poly-ubiquitous degradation of SNAIL protein, which further reversed SNAIL-mediated epithelial-to-mesenchymal transition (EMT) process of HCC cells. In vivo assay by xenograft tumor model verified the antitumor effect of TRIM50 on HCC. Taken together, these results showed that TRIM50 acted as a tumor suppressor in HCC cells by directly targeting SNAIL and reversing EMT, which further indicated that positive modulation of TRIM50 might be a novel therapeutic strategy for SNAIL overexpressed HCC cells.

## Introduction

Hepatocellular carcinoma (HCC) is the primary malignancy of the liver and the third leading cause of cancer-related death worldwide^1–3^. Most of the patients are diagnosed at late stages with limited therapeutic options. Identifying novel disease marker and clarifying the pathological mechanism will provide new insight into this disease and facilitate discovery of novel therapeutic strategies. In recent years, the role of tripartite motif (TRIM) proteins in the development of cancer has attracted much research interest, and novel tumor promoters and tumor suppressors have been identified in TRIM family members^4,5^. TRIM protein family includes >70 highly conserved proteins, which are usually composed of a RING (R) domain, one or two B-boxes (B) domain(s) and a predicted coiled coil (CC) domain^6,7^. TRIM proteins have been reported to play important roles in development, inflammation, anti-virus immunity and cancer^8^. Several TRIM family members were identified to play important roles in the development of liver cancer, which demonstrated that they might have potential applications as novel therapeutic targets or prognostic markers.

Tripartite motif-containing 50 (TRIM50) is a newly identified member of TRIM family and it was first identified as an E3 ubiquitin ligase in Williams–Beuren syndrome^9^. Later reports indicated that TRIM50 promoted the formation of sophisticated canaliculi and microvilli during acid secretion in parietal cells^10^. Another two reports suggested that TRIM50 interacted with HDAC6 and was involved in the regulation of P62 degradation^11,12^. Up to now, reports about the function of TRIM50 is very limited, and its biological function is far from being clarified. The role of TRIM50 in carcinogenesis has never been identified. Because of its recognized E3 ligase activity in diseases, we expected it might be involved in the regulation of the development of HCC.

In the study, we detected the expression of TRIM50 in clinical HCC specimen, analyzed the correlation of TRIM50 expression with disease progression, and further investigated its role in tumor growth, migration, and invasion of HCC cells. All these data revealed that TRIM50 acted as a tumor suppressor in HCC via directly targeting SNAIL and reversing epithelial-to-mesenchymal transition (EMT) process. Thus, this work provided a novel insight into the development of hepatocarcinoma and indicated a novel strategy for the treatment of SNAIL overexpressed HCC cells.

## Results

### TRIM50 was downregulated in HCC tissues and its decreased expression was correlated with advanced disease progression

**Fig. 1:**
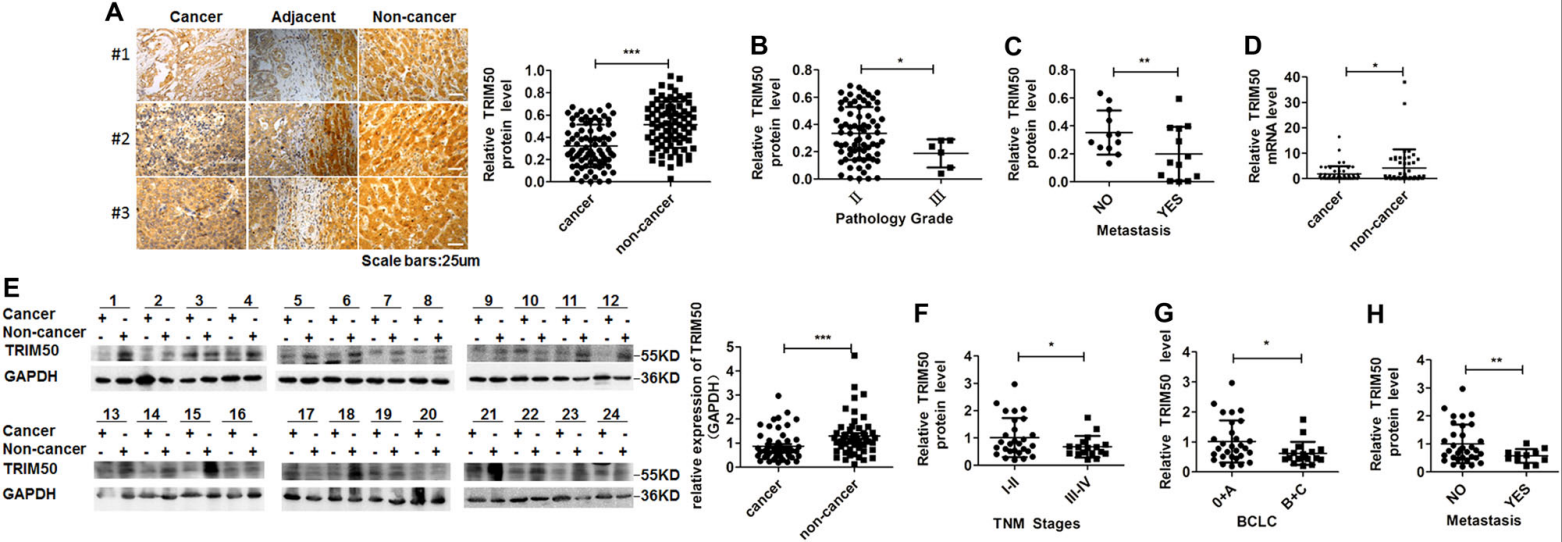
TRIM50 was downregulated in HCC tissues and its expression was inversely correlated with advanced disease progression. **a** Immunohistochemical staining was used to determine the location and expression of TRIM50 in HCC tissues and corresponding non-cancerous liver tissues from 79 clinical HCC patients. The intensities of the IHC staining were quantitatively analyzed by IPP6 software and statistically analyzed (right panel). **b, c** TRIM50 protein levels in different pathology grades (**b**) and different metastasis stages (**c)** from 79 clinical HCC patients used for IHC staining were statistically analyzed and compared. **d** mRNA expression of TRIM50 was determined by qRT-PCR in HCC tissues and corresponding non-cancerous liver tissues from 51 HCC patients. **e** Western blot analysis of protein levels of TRIM50 in the liver cancer tissues and corresponding non-cancerous liver tissues from 52 HCC patients, with GAPDH expression as internal references. The presented images are representative blots from 24 HCC patients. Band intensities of all the investigated patients were measured by Image J software and statistically analyzed (right panel). **f-h** Statistical analysis of TRIM50 protein level in different TNM stages (**f**), different BCLC stages (**g**), and different metastasis stages (**h**) from HCC patients used for western blot assay. *P *<* 0.05, **P < 0.01, and ***P < 0.001 for statistical analysis of the indicated groups

To explore whether expression of TRIM50 in HCC tissues was altered during the development of liver cancer, we detect the levels of TRIM50 in HCC tissues and corresponding non-cancerous liver tissues by immunohistochemistry (IHC), quantitative real-time polymerase chain reaction (qRT-PCR), and western blot. We first detected TRIM50 expression by IHC in HCC tissues and corresponding non-cancerous liver tissues from 79 clinical HCC patients, and our data showed that TRIM50 expression was significantly decreased in the liver cancer tissues compared with corresponding distal non-cancerous liver tissues (Fig. 1a, Supplementary Table 1). To further clarify whether decreased expression of TRIM50 in HCC tissues contributed to disease progression, we further analyzed the correlation between TRIM50 expression and clinical disease status in these IHC staining data. Statistical analysis showed that patients with poorly differentiated tumors, as well as patients with metastasis were prone to have lower levels of TRIM50 expression (Fig. 1b, c).

Then, we did qRT-PCR assay in a cohort of 51 HCC patients and western blot assay in another cohort of 52 HCC patients. Both the qRT-PCR data (Fig. 1d) and western blot data (Fig. 1e) verified the IHC data, which showed that TRIM50 expression was significantly decreased in HCC tissues compared with corresponding non-cancerous liver tissues. Further assay of western blot data showed that patients with advanced Tumor Lymph Node Metastasis stages (TNM stages), Barcelona Clinic Liver Cancer stages (BCLC stages) and metastasis were prone to have lower levels of TRIM50 expression (Fig. 1f, h). Altogether, these data indicated that TRIM50 was downregulated in HCC tissues and its decreased expression contributed to HCC progression.

### TRIM50 inhibited proliferation, colony formation, and invasion of HCC cells

**Fig. 2:**
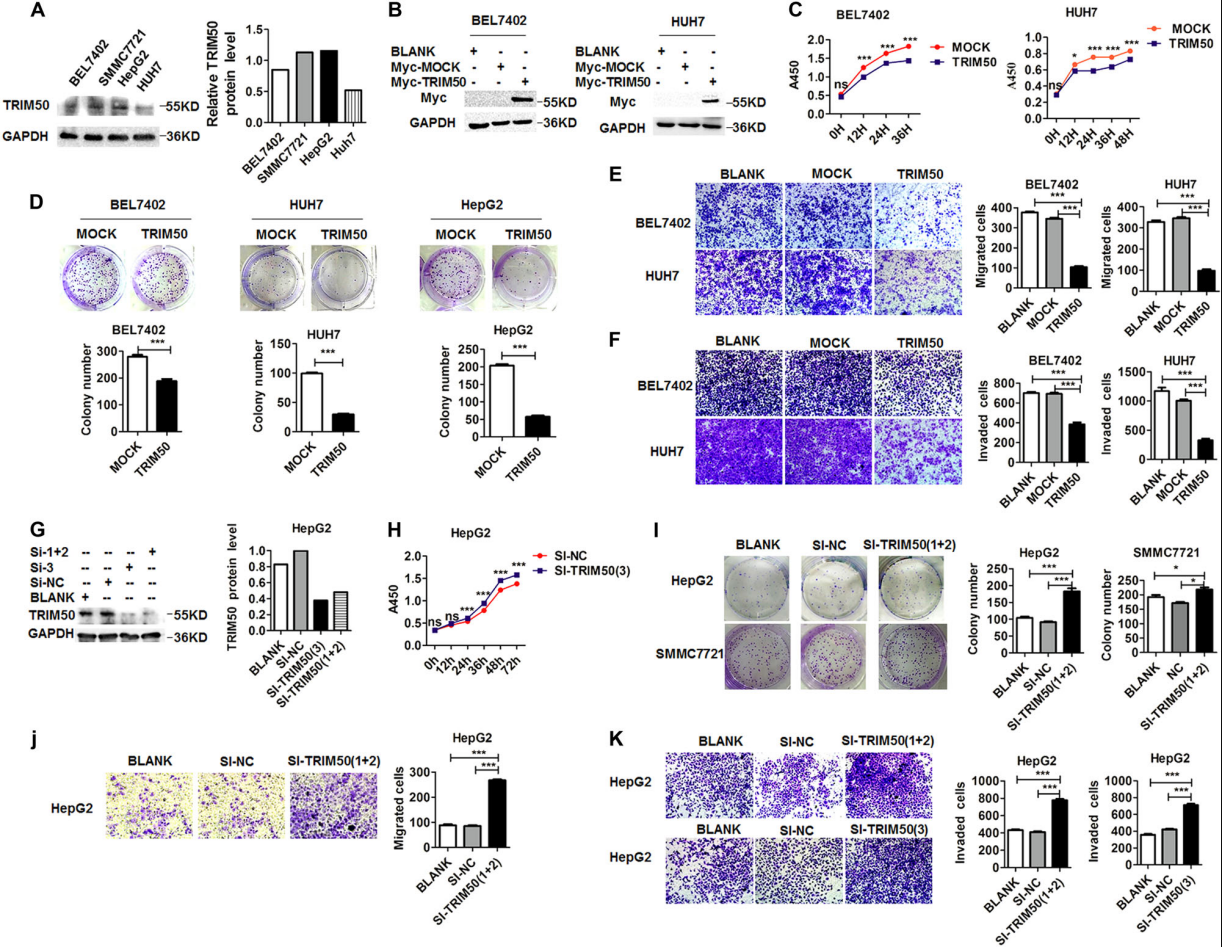
TRIM50 inhibited proliferation, colony formation, and invasion of HCC cells. **a** The basic protein levels of TRIM50 in BEL7402, SMMC7721, HepG2, and HUH7 cells were detected by western blot. **b** BEL7402 and HUH7 cells were transfected with TIRM50 expression plasmid or mock control, and western blot assay was performed to define the successful exogenous overexpression of TRIM50 in HCC cells. **c** BEL7402 and HUH7 cells were transfected with TRIM50 plasmid or mock control, and proliferation status of the transfected HCC cells was detected at 0 h, 12 h, 24 h, 36 h, and 48 h by CCK8 assay. **d** BEL7402, HUH7, and HepG2 cells were transfected with TRIM50 expression plasmid or mock control, and further cultured for 24 h before being transferred to six-well plates at the density of 1000 cells per well for colony formation assay. The clone formations were harvested after 14 days and the number of clone formation was counted. **e, f** After transfection with TRIM50 plasmid or mock control, transwell migration assay (**e)** and transwell invasion assay (**f**) were performed to investigate the migration and invasion capabilities of HCC cells. **g** HepG2 cells were transfected with siRNAs specifically targeting TRIM50 (Si-TRIM50-1&2, and Si-TRIM50-3), the cells transfected with random sequences (Si-NC) were used as mock control. The cells were further cultured for 24 h before being harvested and the block efficiency was measured by western blot. **h** HepG2 cells were transfected with siRNAs specifically targeting TRIM50 (Si-TRIM50-1&2) or its nonsense control (Si-NC), and proliferation status of transfected HCC cells were measured at 0 h,12 h, 24 h, 36 h, and 48 h by CCK8 assay. **i** HepG2 cells and SMMC7721 cells were transfected with Si-TRIM50-1&2 or Si-NC and further cultured for 24 h. The transfected cells were further transferred to six-well plates at the density of 1000 cells per well and allowed to grow for 14 days for colony formation assay. **j, k** After transfection of Si-TRIM50 or nonsense control (Si-NC) to HepG2 cells, transwell migratory assay (**j)** and invasion assay (**k**) of HCC cells were performed. Similar results were obtained in at least three independent experiments. *P *<* 0.05 and ***P < 0.001 for statistical analysis of the indicated groups

To explore the effect of TRIM50 on the malignant behaviors of HCC cells, we constructed gain-of-function model by transfection of TRIM50 into HCC cells, and loss-of-function model by transfection of small interference RNA against TRIM50 into HCC cells. Western blot data showed that the protein levels of TRIM50 were lower in BEL7402 cells and HUH7 cells compared with those in HepG2 cells and SMMC7721 cells (Fig. 2a). Thus, BEL7402 and HUH7 cells were transfected with TRIM50 plasmid to construct the gain-of-function cellular model; and HepG2 cells and SMMC7721 cells were transfected with small interference RNA against TRIM50 (Si-TRIM50) to construct the loss-of-function model. These cellular models were investigated to define the effect of TRIM50 on the malignant behaviors of HCC cells.

Our data showed that after successful overexpression of TRIM50 in HCC cells (Fig. 2b), proliferation, colony formation, migration, and invasion capabilities of HCC cells were significantly inhibited (Fig. 2c-f). After successful knockdown of TRIM50 expression by its specific siRNAs (Fig. 2g), the proliferation, colony formation, migration, and invasion capabilities of HCC cells were significantly increased (Fig. 2h-k). Altogether, both of our gain-of-function model and loss-of-function model support the conclusion that TRIM50 could act as a tumor suppressor to inhibit malignant behaviors of HCC cells.

### TRIM50 reversed resistance to anoikis of HCC cells

Resistance to anoikis is the hallmark of cancer and the prerequisite step for distant metastasis of HCC cells. Our previous data showed that HCC cells resisted to anoikis after anchorage deprival and acquired more malignant properties during its anoikis-resistant process^13,14^. In this study, we are interested to know whether TRIM50 also plays a role in the resistance to anoikis of HCC cells. Our data showed that TRIM50 overexpression significantly decreased cell viabilities in the anchorage-deprived HCC cells, which indicated that TRIM50 could reverse resistance to anoikis of HCC cells (Supplementary Figure 1A). Caspase cascade assay further verified that TRIM50 reversed anoikis resistance of HCC cells and induced apoptotic cell death after anchorage deprival (Supplementary Figure 1B and 1C).

Our previous data showed that during the process of anoikis resistance, the malignant behaviors of HCC cells were also significantly increased^13,14^. Thus, we are interested to verify whether these malignant behaviors of anoikis-resistant HCC cells could also be influenced by TRIM50. Our data showed that TRIM50 overexpression significantly inhibited the colony formation and invasion capabilities of anchorage-deprived HCC cells (Supplementary Figure 1D and E). These data further support the role of TRIM50 in HCC cells as a tumor suppressor.

### TRIM50 exerted its antitumor effect through directly targeting SNAIL and reversing EMT

**Fig. 3:**
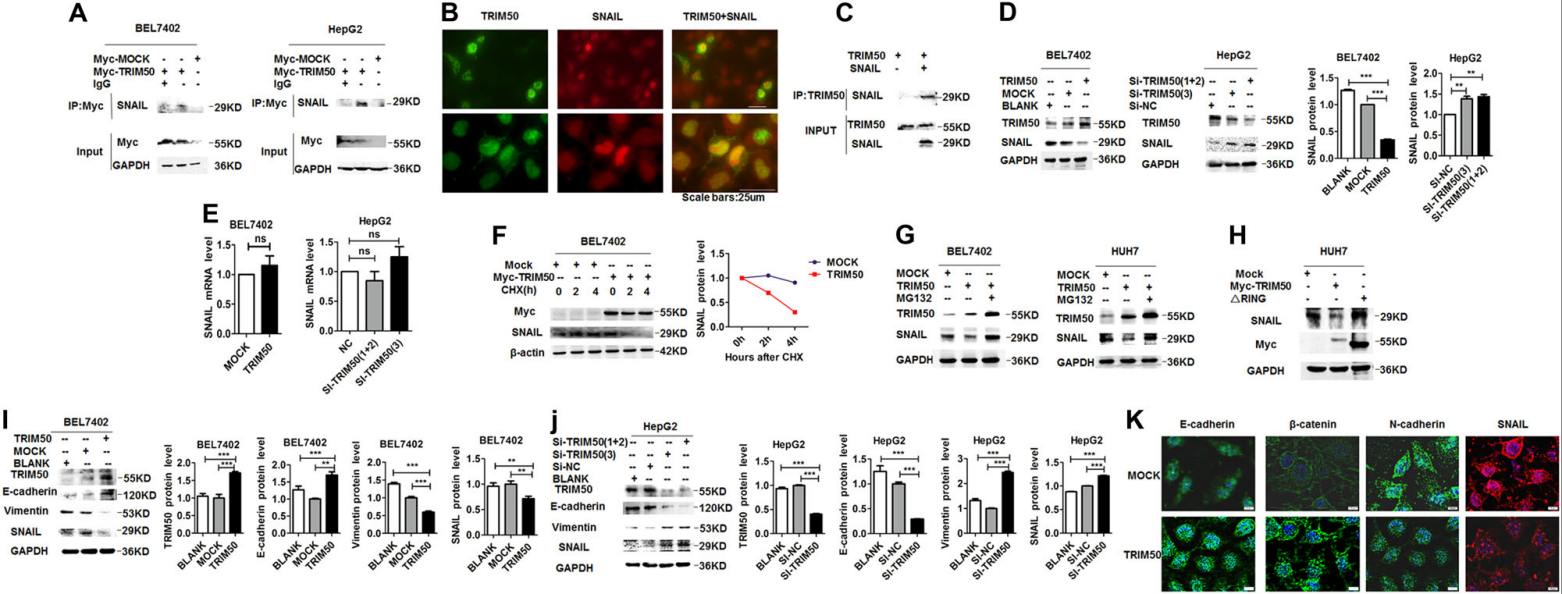
TRIM50 exerted its antitumor effect through directly targeting SNAIL and reversing EMT. **a** BEL7402 and HepG2 cells were transfected with TRIM50 plasmid or mock control, the binding between TRIM50 and SNAIL protein were detected by immunoprecipitation. **b** HCC cells were cultured for 24 h before immunofluorescence assay to detect the expression and colocalization status of TRIM50 (green) and SNAIL (red). **c** TRIM50 and SNAIL proteins were separately expressed by the in vitro transcription and translation system, and the direct binding between TRIM50 and SNAIL were analyzed by co-IP assay. **d, e** BEL7402 cells were transfected with TRIM50 plasmid or mock control, and HepG2 cells were transfected Si-TRIM50 or Si-NC, the transfected cells were further cultured for 24 h. **e** The protein level of SNAIL was detected by western blot and further quantitatively analyzed (**d**); and the mRNA level of SNAIL was detected by qRT-PCR (**e**). **f** BEL7402 cells were transfected with TRIM50 plasmid or mock control and further cultured for 24 h before cyclohexamide (CHX) was put into the transfected cells. The cells were further cultured for 0 h, 2 h, and 4 h before being harvested for western blot assay of SNAIL expression. The band intensities were further quantitatively analyzed (right panel). **g** BEL7402 cells and HUH7 cells were transfected with TRIM50 plasmid or mock control, and further cultured for 24 h. Transfected cells were treated with MG132 (10 μM) for 4 h before protein lysates were isolated to detect the expression level of TRIM50 and SNAIL by western blot. **h** Wild-type Myc-tagged TRIM50 or its RING domain deleted mutant(△RING) was co-transfected with SNAIL into HUH cells, and further cultured for 24 h before being harvested for western blot assay of SNAIL. **i** BEL7402 cells were transfected with TRIM50 plasmid or mock control, and further cultured for 24 h. The expression of E-cadherin, vimentin, and SNAIL was detected by western blot and quantitatively analyzed. **j** HepG2 cells were transfected with Si-TRIM50 or Si-NC, and further cultured for 24 h. The expression of E-cadherin, vimentin, and SNAIL was detected by western blot and quantitatively analyzed. **k** BEL7402 cells were transfected with TRIM50 plasmid or mock control, and the cells were further cultured for 24 h before immunofluorescence assay to detect the expression of E-cadherin, β-catenin, N-cadherin, and SNAIL. Similar results were obtained in at least three independent experiments. **P < 0.01 and ***P < 0.001 for statistical analysis of the indicated groups

TRIM family members usually take their effects by direct binding with target proteins and exert their function through modulation of target molecules. To further define the molecular mechanism of TRIM50 in the regulation of HCC progression, we tested a series of molecules, which might be involved in the process of carcinogenesis to define the target of TRIM50 by immunoprecipitation (data not shown). Our immunoprecipitation data showed that TRIM50 could bind with SNAIL protein (Fig. 3a). Further immunofluorescence (IF) data showed that TRIM50 and SNAIL could colocalized in HCC cells (Fig. 3b), which indicated the interaction between TRIM50 and SNAIL in HCC cells. To further define whether the interaction between TRIM50 and SNAIL is a direct binding effect, we did immunoprecipitation assay with an in vitro transcription and translation system as described before^15^. Our data showed that TRIM50 protein could directly interact with SNAIL protein as detected by the in vitro translation system (Fig. 3c), which indicated that TRIM50 could bind with SNAIL protein directly. Thus, these data indicated that TRIM50 might exert its function through its direct interaction and modulation of SNAIL. To further define whether TRIM50 could have any influence on the expression of SNAIL, we detected the protein levels of SNAIL in TRIM50 overexpressed cells and TRIM50 knockdown cells by western blot and mRNA level of TRIM50 by real-time PCR. Our data verified that TRIM50 could negatively regulate SNAIL expression at the protein level but not at the mRNA level (Fig. 3d, e). This negative regulation of SNAIL by TRIM50 was also verified in the clinical HCC patients (Supplementary Figure 2). Further cyclohexamide (CHX) chase assay showed that TRIM50 increased the degradation of SNAIL after de novo protein synthesis was blocked (Fig. 3f). When we put proteasome inhibitor MG132 in HCC cells, the negative regulation of SNAIL by TRIM50 was significantly rescued, which indicated that TRIM50 regulated SNAIL by proteasome mediated degradation (Fig. 3g). The presence of RING domain confers E3 ligase activity to TRIM family members, thus we were interested to know whether RING domain was responsible for the negative regulation of SNAIL by TRIM50. Therefore, we transfected HUH7 with TRIM50 RING domain mutant and analyzed its effect on the expression of SNAIL. Our data showed that deletion of RING domain in TRIM50 significantly rescued the negative regulation of SNAIL by TRIM50 (Fig. 3h). Thus, these data indicated that TRIM50 negatively regulated SNAIL expression via its RING domain.

SNAIL is recognized as a transcription factor, which plays a critical role in the regulation of EMT process and further promotes the development of cancer. It is reported that suppression of E-cadherin is a key step in EMT process, whereas SNAIL is reported to direct repress E-cadherin^16^. Thus, SNAIL-mediated E-cadherin repression is a critical step in the EMT process of cancer^17^. As our data showed the effective suppression of SNAIL by TRIM50 in HCC cells, we are further interested to define whether TRIM50 acts as a tumor suppressor through its suppression of SNAIL-mediated EMT process. Our data showed that when HCC cells were transfected with TRIM50 plasmid, with the suppression of SNAIL expression, expression of the epithelia marker E-cadherin was significantly upregulated, whereas expression of the mesenchyme marker vimentin was significantly downregulated (Fig. 3i). When TRIM50 expression was blocked by its specific interference RNAs, expression of E-cadherin was significantly downregulated, whereas vimentin level was significantly upregulated (Fig. 3j). Further IF assay confirmed the positive regulation of E-cadherin and β-catenin by TRIM50, and negative regulation of N-cadherin and SNAIL by TRIM50 (Fig. 3k). Besides, the phenotypic changes of HCC cells after overexpression of TIRM50 also indicated negative regulation of the EMT process by TRIM50 (Supplementary Figure 3). These data verified that TRIM50 acted as a tumor suppressor through its negative regulation of SNAIL and further reversing the EMT process.

### TRIM50 induced ubiquitous degradation of SNAIL by K-48 linked poly-ubiquitination

**Fig. 4:**
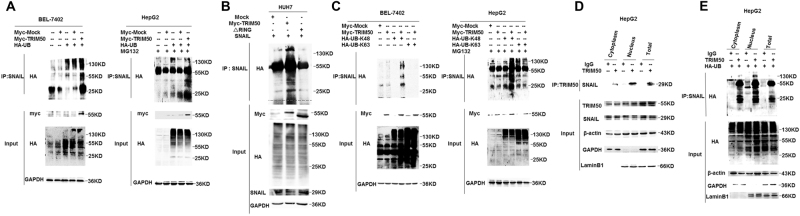
TRIM50 induced ubiquitous degradation of SNAIL by K-48 linked poly-ubiquitination. **a** BEL7402 cells and HepG2 cells were transfected with TRIM50 plasmid and/or HA-UB plasmid as indicated, and further cultured for 24 h. The ubiquitous status of SNAIL was analyzed by co-IP assay. **b** HUH cells were co-transfected with Myc-tagged TRIM50 plasmid or TRIM50 truncation mutant (△RING) together with SNAIL expression plasmid, and further cultured for 24 h. The ubiquitous status of SNAIL was analyzed by co-IP assay. **c** BEL7402 cells and HepG2 cells were co-transfected with TRIM50 and HA-K-48-UB/HA-K-63-UB plasmids, and the ubiquitous status of SNAIL was analyzed by co-IP. **d** Cytoplasmic and nucleic fractions of HCC cells were prepared from HepG2 cells, and co-IP assay was used to detect the interaction between TRIM50 and SNAIL in different fractions of HCC cells. **e** The ubiquitous status of SNAIL protein in different fraction of HCC cells was also detected by co-IP. Lamin B1 was used as nuclear internal control, GAPDH was used as cytoplasm control, and β-actin was served as whole-cell loading control. Similar results were obtained in at least three independent experiments

Based on the presence of typical RING domain in TRIM50, we speculated that TRIM50 might exert its E3 ligase activities on SNAIL via its RING domain. Thus, we co-transfected the HCC cells with TRIM50 plasmid and HA-UB plasmid, followed by the immunoprecipitation assay to verify whether TRIM50 could put the poly-ubiquitin chain to SNAIL. Our data showed that at the presence of TRIM50, the poly-ubiquitin chain was successfully put to SNAIL protein, which indicated that TRIM50 regulated SNAIL by its poly-ubiquitous modification of SNAIL protein (Fig. 4a). Further assay showed that RING domain deleted TRIM50 mutant failed to put the poly-ubiquitin chain to SNAIL, which indicated that TRIM50 regulated ubiquitous modification of SNAIL via its RING domain (Fig. 4b). Lysine-48 (K-48)-linked poly-ubiquitous modification is mainly involved in targeting proteins for proteasomal degradation, whereas Lysine-63 (K-63)-linked poly-ubiquitous modification is coupled to mediate non-proteolytic signals, including those regulating subcellular localization, protein activation, and protein interactions^18^. By co-transfection of SNAIL and K-63-only or K-48-only ubiquitin constructs into HCC cells, we found that TRIM50 could put the K-48 linked but not K-63 linked poly-ubiquitin chain to SNAIL protein (Fig. 4c). These data suggested that TRIM50 acted as an E3 ubiquitin ligase and mediated K-48 linked poly-ubiquitous degradation of SNAIL protein.

To further clarify whether TRIM50-mediated ubiquitous degradation of SNAIL occurred in the nuclear or cytoplasmic compartment, we isolated different compartments of the HCC cells for further analysis. Our immunoprecipitation data showed that TRIM50 could bind with SNAIL in both the nuclear and cytoplasm (Fig. 4d). When we co-transfected TRIM50 and HA-UB plasmid into HCC cells, our data showed that TRIM50 could successfully put the poly-ubiquitin chain to SNAIL in both the nucleic and cytoplasmic compartments. These data indicated that TRIM50 mediated ubiquitous degradation of SNAIL in the cytoplasmic, as well as in the nucleic compartment (Fig. 4e). Collectively, these above data also support our previous results showing that TRIM50 induced K-48 linked poly-ubiquitous degradation of SNAIL in HCC cells.

### Exogenous overexpression of SNAIL rescued the antitumor effect of TRIM50

**Fig. 5:**
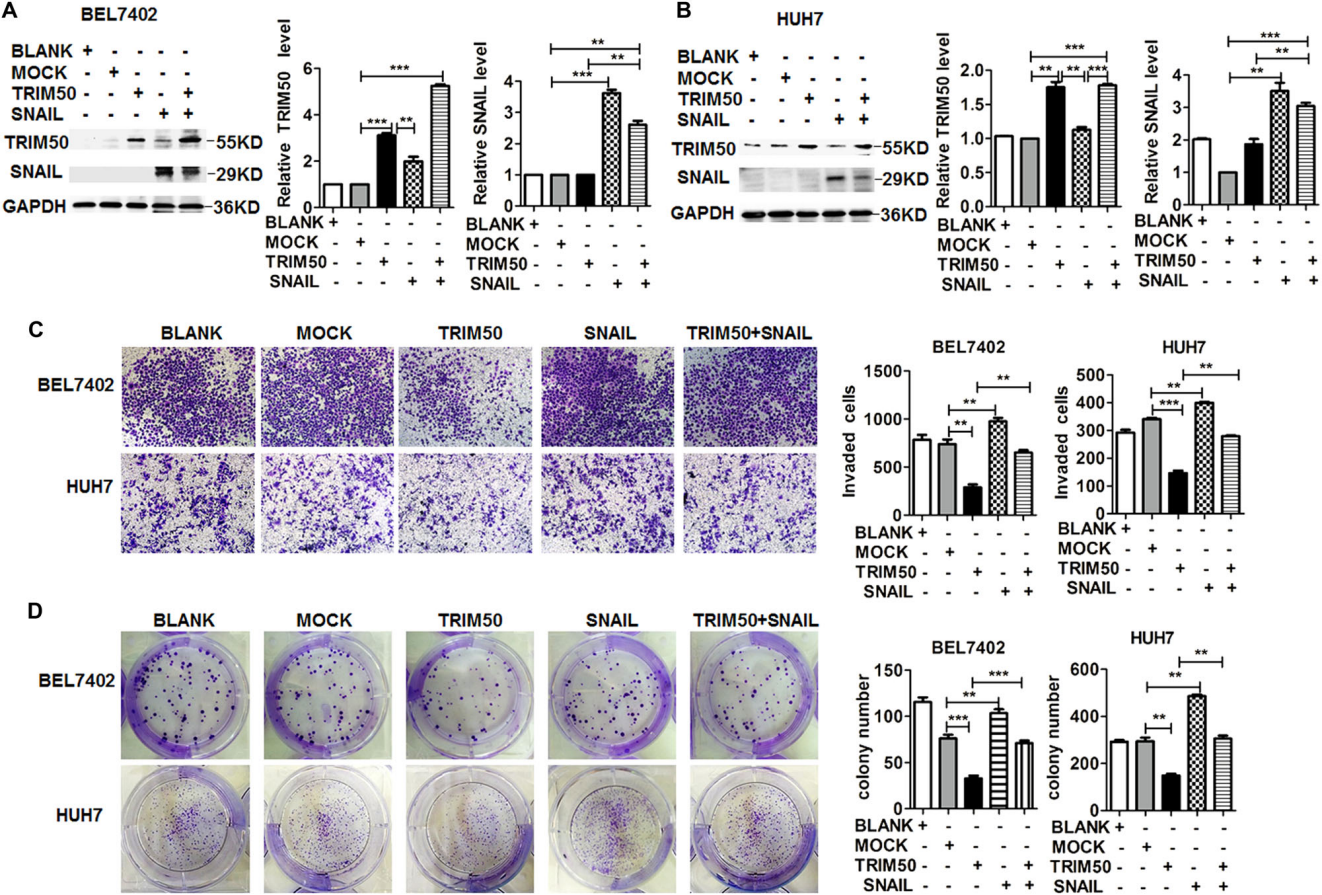
Exogenous overexpression of SNAIL significantly rescued the antitumor effect of TRIM50. **a, b** BEL7402 cells (**a**) and HUH7 cells (**b**) were transfected with TRIM50 plasmid and SNAIL plasmid, and the expression of TRIM50 and SNAIL were detected by western blot. **c** BEL7402 and HUH7 cells were transfected with TRIM50 plasmid or SNAIL plasmid, and transwell invasive assay was performed to detect the invasive capability of these transfected HCC cells. **d** BEL7402 cells were transfected with TIRM50 expression plasmid or SNAIL expression plasmid, and the cells were transferred to six-well plates at the density of 1000 cells per well for colony formation assay. The colonies were stained after 14 days, and the number of colonies was counted and statistically analyzed. **P < 0.01 and ***P < 0.001 for statistical analysis of the indicated groups

Our data showed that TRIM50 directly targeted SNAIL for degradation and further inhibited malignant behaviors of HCC cells. Thus, we are interested to know whether overexpression of SNAIL could rescue the tumor-suppressor role of TRIM50. We co-transfected TRIM50 and SNAIL plasmid into HCC cells, and western blot assay verified successful overexpression of both TRIM50 and SNAIL proteins (Fig. 5a, b). Further assay showed that inhibition of malignant behaviors of HCC cells by TRIM50 overexpression was significantly reversed after transfection with SNAIL plasmid (Fig. 5c, d). These data further confirmed that TRIM50 in these HCC cells prohibited cancer progression through directly targeting SNAIL for degradation.

### Xenografted tumor model verified the antitumor effect of TRIM50

**Fig. 6:**
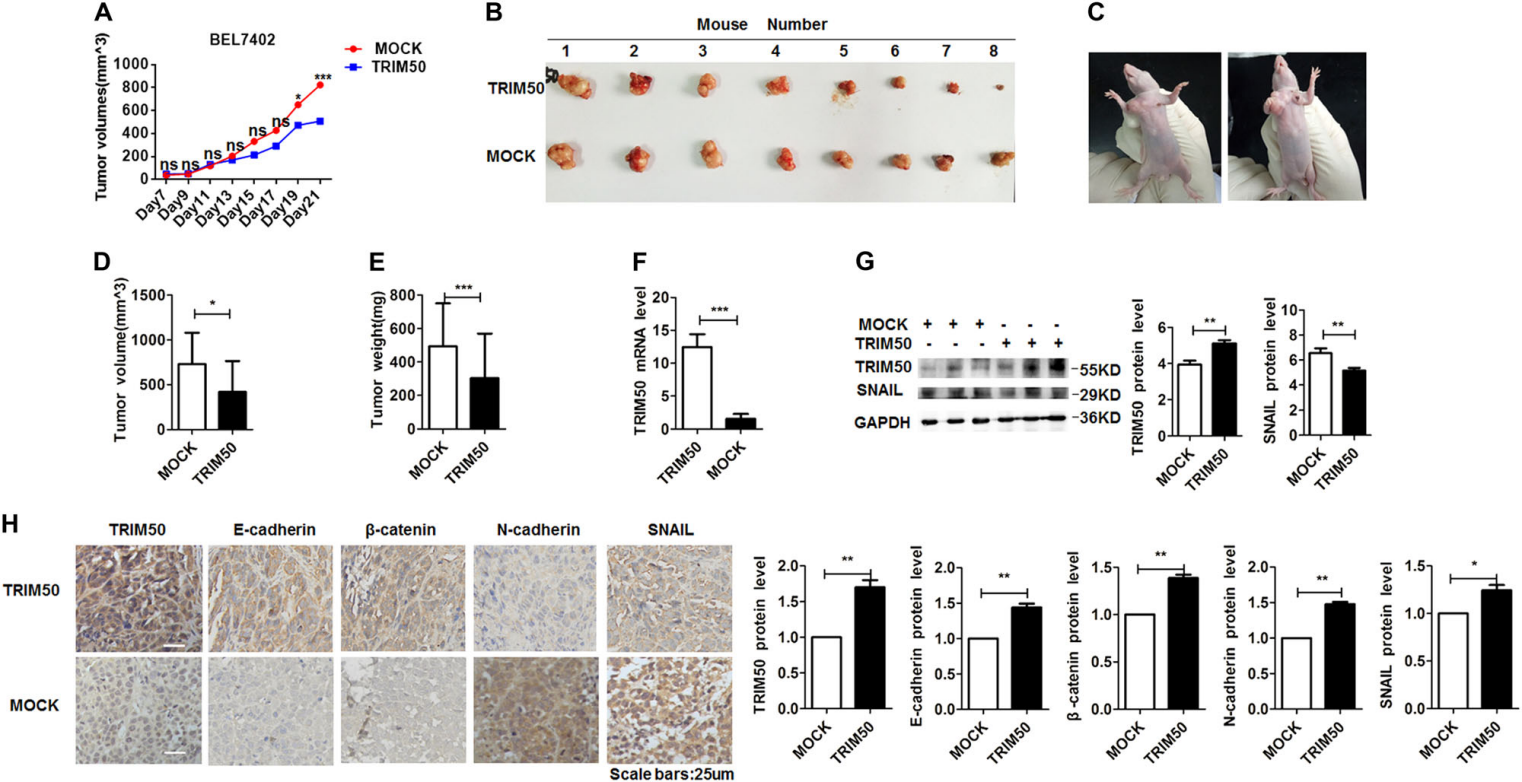
Antitumor effect of TRIM50 was verified in xenografted tumor model. BEL7402 cells (10^7^ cells) were subcutaneously injected to both flanks of the nude mice. When visible tumor appeared, TRIM50 expression plasmid was injected to the formed tumor in the left flank and mock control plasmid was injected to the tumor in the right flank every other day before the mice were sacrificed on day 28. **a** The growth curves of tumors with TRIM50 or mock plasmid transfection were analyzed every other day before the mice were sacrificed. **b** The formed tumors with TRIM50 or mock plasmid transfection were isolated and compared. **c** Images presented were the representative mice with subcutaneous xenograft tumor. **d, e** The volume (**d**) and weight (**e**) of the formed tumors transfected with TRIM50 plasmid or mock plasmid were analyzed and compared. **f** mRNA levels of TRIM50 in TRIM50 or mock control transfected tumors were analyzed by qRT-PCR. **g** Western blot assay was performed to detect the protein levels of TRIM50 and SNAIL in the formed tumors. **h** Immunohistochemical staining was used to detect the level of TRIM50, E-cadherin, β-catenin, N-cadherin, and SNAIL in formed tumors. *P *<* 0.05, **P < 0.01, and ***P < 0.001 for statistical analysis of the indicated groups

To further assess the antitumor effect of TRIM50 in vivo, we constructed xenograft tumor models by injection of BEL7402 cells to both flanks of nude mice. When visible tumor appeared, we injected TRIM50 expression plasmid to the left flanks and mock control to the right flanks of the mice. These plasmids were injected to the formed tumor every other day, and the sizes of the formed tumor were also measured until the mice were sacrificed on day 28 after the transplantation. The growth kinetics of the formed tumor showed that transfection of TRIM50 significantly inhibited tumor growth (Fig. 6a). The excised tumors from each group were compared, which showed that TRIM50 overexpressed tumors were much smaller than the mock group (Fig. 6b, c). The average size (d) and weight (e) of the TRIM50 transfected tumors were significantly decreased compared with the mock control group (Fig. 6d, e). qRT-PCR, IHC, and western blot assay further verified that TRIM50 was successfully overexpressed in the TRIM50 plasmid transfected group (Fig. 6f-h). Western blot assay confirmed that SNAIL expression was significantly suppressed in the TRIM50 transfected tumors (Fig. 6g). IHC assay confirmed the positive regulation of E-cadherin and β-catenin by TRIM50, and negative regulation of N-cadherin and SNAIL by TRIM50 (Fig. 6h). Thus, these in vivo data further verified our in vitro data that TRIM50 inhibited HCC growth through it suppression of SNAIL.

## Discussion

Recent studies indicated that several members of the TRIM protein family were important regulators of carcinogenesis. Among these, TRIM24, TRIM26 were identified as tumor suppressors in the development of HCC, whereas TRIM31 was identified as a tumor promoter for HCC^5,15,19^. However, the role of TRIM50 in the progression of HCC was unknown. In this study, we identified the tumor-suppressor role of TRIM50 in the development of HCC and further clarified its involved molecular mechanism for the first time.

We first investigated the expression of TRIM50 in clinical specimen, and all of our data showed a significantly decreased expression of TRIM50 in HCC tissues, and its expression was inversely correlated with clinical stages and differentiation status of the patients. These data indicated that decreased expression of TRIM50 may facilitate the development of liver cancer. Further cellular model data showed that proliferation, colony formation, and invasion capabilities of HCC cells were significantly inhibited after ectopic overexpression of TRIM50 in HCC cells, whereas these malignant behaviors were significantly enhanced after block of TRIM50 in HCC cells. These data indicated the tumor-suppressor role of TRIM50 in HCC cells, and further suggested that loss of TRIM50 in HCC tissues could lead to the progression of liver cancer.

Recent reports showed the function of TRIM proteins often depended on their interactions with other proteins, usually target proteins^20^. In this study, we identified SNAIL as a novel binding partner of TRIM50 in liver cancer cells. At the cellular level, we demonstrated that TRIM50 negatively regulated SNAIL expression. Further investigation showed that TRIM50 could directly bind with SNAIL and induce K-48 linked poly-ubiquitination of SNAIL protein. To further clarify whether decreased expression of SNAIL by TRIM50 was required for TRIM50-induced antitumor effect on HCC cells, we re-introduced SNAIL into TRIM50 overexpressed cells and measured its influence on the malignant behaviors of HCC cells. Our data showed that ectopic overexpression of SNAIL significantly rescued the antitumor effect of TRIM50, which further verified that TRIM50 exerted its effect on HCC cells through its negative regulation of SNAIL.

SNAIL is a conserved transcription factor playing an essential role in EMT during cancer metastasis. EMT is a critical process involved in cancer progression. A hallmark for EMT is the loss of cell adhesion molecule E-cadherin, and it is reported that SNAIL could directly repress E-cadherin^16,21,22^. Our data showed that overexpression of TRIM50 in HCC cells could increase E-cadherin expression, which indicated that TRIM50 might exert its antitumor effect through reversing SNAIL-mediated EMT process.

**Fig. 7:**
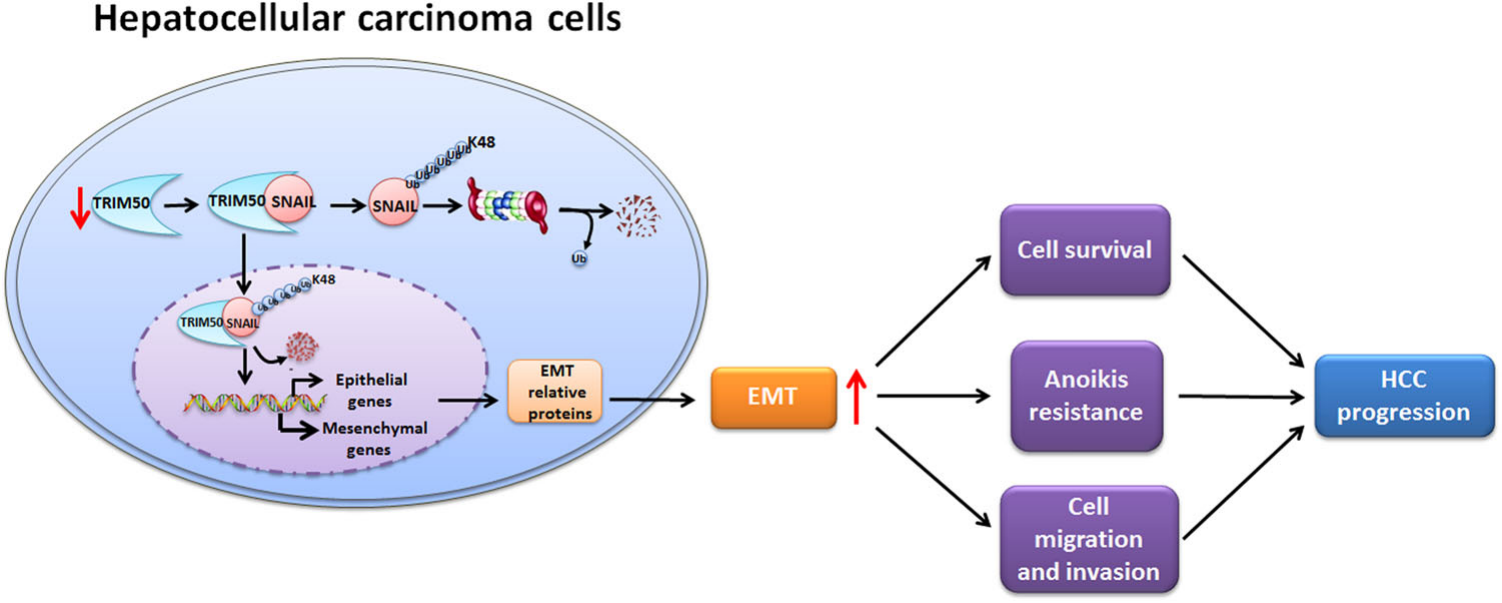
Working model of the role of TRIM50 in HCC progression. TRIM50 was significantly downregulated in HCC cells and its decreased expression further promoted HCC progression. Further investigation showed that TRIM50 could target SNAIL for K-48 linked poly-ubiquitous degradation and thus reversed SNAIL-mediated epithelial-to-mesenchymal transition (EMT) transition. Altogether, loss of TRIM50 in HCC cells led to upregulation of EMT process and further promoted the malignant behaviors of HCC cells, including proliferation, colony formation, anoikis resistance, and invasion, thus promoted HCC progression

It is reported that SNAIL is expressed in both the cytoplasm and nuclear in cancer cells^16,23^. To further clarify the interaction between TRIM50 and SNAIL, we separated different compartments of HCC cells and did the immunoprecipitation assay to identify where this interaction occurred. Our data showed that TRIM50 could bind with SNAIL in both the cytoplasmic and nucleic compartments of HCC cells (Fig. 7). These data indicated that TRIM50 could act as a tumor suppressor by directly targeting SNAIL in both cytoplasmic and nuclear compartments of cancer cells.

Like other TRIM family members, TRIM50 has a typical RING domain, which may confer it ubiquitous activation to its target proteins^24^. Ubiquitination is one of the most abundant and versatile post-translation modifications in cells where the ubiquitin is covalently added to lysine residues of target protein. There are several types of ubiquitin modifications with different effects on target proteins. For instance, the K-48 linked poly-ubiquitination could induce ubiquitous degradation of target proteins, whereas the K-63 linked poly-ubiquitination could modulate the activation of target proteins^18,25^. To better understand the posttranslational regulation of SNAIL by TRIM50, we performed the immunoprecipitation assay by co-transfection of SNAIL and ubiquitin expression plasmids into HCC cells. Our data showed that TRIM50 could successfully put the poly-ubiquitin chain to SNAIL. Further analysis showed that TRIM50 could induce K-48 linked, but not K-63 linked poly-ubiquitination of SNAIL protein. Thus, we identified SNAIL as a novel important target for TRIM50-mediated poly-ubiquitination, and further analysis verified that TRIM50 induced K-48 linked ubiquitous degradation of SNAIL.

In conclusion, we investigated the role of TRIM50 in HCC progression in an integrate investigation system including clinical specimen, cellular model, and animal model. Our study showed that TRIM50 expression was significantly decreased in HCC tissues compared with corresponding distal non-cancerous tissues. Its downregulation was significantly inversely correlated with disease progression, which indicated its involvement in the development of cancer. Further in vitro and in vivo study verified the antitumor effect of TRIM50 on HCC cells was mediated by its K-48 linked poly-ubiquitous degradation of SNAIL protein. Altogether, this study provided clues to understand the pathogenesis of HCC, and it indicated that therapeutic strategy by upregulating TRIM50 in SNAIL overexpressed cancers may pave a new avenue for manipulating liver cancer.

## Materials and methods

### Tissue samples

Paired samples of HCC tissues and corresponding non-cancerous liver tissues from the Department of Hepatobiliary Surgery of the Provincial Hospital Affiliated to Shandong University were used for detection of TRIM50 expression. Among them, 79 pairs of liver cancer and corresponding non-cancerous tissues were used for IHC assay, 52 pairs of matched specimens were used for western blot assay, and 51 pairs of matched specimens were used for qRT-PCR. These procedures dealing with human specimen were approved by Shandong University Research Ethics Committee and all the protocols dealing with the patients met the ethical guidelines of the Helsinki Declaration. Written informed consent was obtained from each patient before participation and approved by the ethics committee of Shandong University. Details of the clinicopathologic characteristics of these recruited HCC patients were shown in Table 1.

**Table 1:** Clinicalpathological characteristics of the investigated HCC patients

	Cohort 1 (*n* = 79)	Cohort 2 (*n* = 51)	Cohort 3 (*n* = 52)
	(for IHC)	(for qRT-PCR)	(for western blot)
Gender			
Male	68 (86.1%)	40 (78.4%)	42 (80.8%)
Female	11 (13.9%)	11 (21.6%)	10 (19.2%)
Age			
<54	32 (40.5%)	19 (32.3%)	21 (40.4%)
≥54	47 (59.5%)	32 (62.7%)	31 (59.6%)
Tumor size			
<5 cm	48 (60.7%)	28 (54.9%)	16 (30.8 %)
≥5 cm	31 (39.3%)	23 (45.1%)	36 (69.2%)
Liver cirrhosis history			
Yes	65 (82.3%)	34 (66.7%)	32 (62.7%)
No	14 (17.7%)	17 (33.3%)	20 (39.3%)
TNM stages			
I	38 (48.1%)	22 (43.1%)	23 (44.2%)
II	2 (2.5%)	5 (9.8%)	3 (5.8%)
III	32 (40.5%)	14 (27.5%)	16 (30.8%)
IV	5 (6.3%)	7 (13.7%)	5 (9.6%)
Not available	2 (2.5%)	3 (5.9%)	5 (9.6%)
Regional lymph nodes			
N0	73 (92.4%)	44 (86.3%)	44 (84.6%)
N1	6 (7.6%)	7 (13.7%)	8 (15.4%)
BCLC stages			
0	6 (7.6%)	1 (2.0%)	11 (21.2%)
A	30 (38.0%)	10 (19.6%)	18 (34.6%)
B	10 (12.6%)	4 (7.8%)	8 (15.4%)
C	24 (30.4%)	15 (29.4%)	10 (19.2%)
D	2 (2.5%)	0	0
Not available	7 (8.9%)	21 (41.2%)	5 (9.6%)
Distant metastasis			
No	13 (16.5%)	46 (90.2%)	33 (63.5%)
Yes	12 (15.4%)	3 (5.9%)	11 (21.2%)
Not available	54 (68.1%)	2 (3.9%)	8 (15.3%)

### Immunohistochemistry

IHC was performed to detect the expression and location of TRIM50 on paraffin sections of HCC tissues and non-cancerous liver tissues. IHC staining and evaluation were performed according to the procedure described before^26,27^. Specific antibody against TRIM50 (ab174880) was from Abcam company (Cambridge, MA, USA). Immunohistochemical staining was evaluated using Image-Pro Plus v6.2 software (Media Cybernetics, Inc., Bethesda, MD, USA). For accurate reading of the staining, we used the same setting for all the analyzed fields. Integrated optical density (IOD) was measured in all investigated fields, and density of positive staining was evaluated as IOD/the total area of each field.

### Quantitative real-time PCR

Total RNA was extracted from liver cancer tissues and qRT-PCR was performed as described before^15,27^. Primers for human TRIM50 gene were forward: 5′-CCCATTTGCCTGGAGGTCTTC-3′, reverse: 5′-CAGGACAGCATAGCTCGGAG-3′. Relative gene expression levels were normalized to β-actin. Primers for β-actin gene were forward: 5′-GGCACCACACCTTCTACAATG-3′, reverse: 5′-TAGCACAGCCTGGATAGCAAC-3′.

The relative mRNA levels of target genes were obtained by using the 2 ^–ΔΔ^Ct method with all assays performed in triplicate.

### Cell culture, transfection and IF

All of the HCC cell lines, including BEL7402, SMMC7721, HepG2 and HUH7 cells, were obtained and cultured as previously described^15^. HCC cells grown on normal plates and poly-2-hydroxyethylmethacrylate (poly-HEMA) coated plates were established as attached cell and detached anchorage-deprived cells, respectively^13,14,28^. TRIM50 plasmid was synthesized by OriGene (OriGene Technologies, Maryland, USA). RING domain deleted mutant of TRIM50 was generated using the KOD-Plus-Mutagenesis kit (Toyobo, Osaka, Japan) according to the manufacture’s protocol. The small interfering RNAs targeting TRIM50 and SNAIL were synthesized by RIBOBIO (RIBOBIO, Guangzhou, China). Transfection and IF assay were performed as previously describe^15^.

### Western blot and co-immunoprecipitation (co-IP)assay

Western blot and co-IP were performed as described before^13,28^. The primary antibodies used in these assays included antibodies against TRIM50 (ab174880, Abcam, Cambridge, MA, USA), SNAIL (13099-1-AP, Proteintech, China), E-cadherin (610181, BD Biosciences, San Jose, CA), Vimentin (610916, Cell Signaling Technology, Beverly, USA), β-actin (TA-09, Proteintech, Chicago, USA), and MYC (TA150121, OriGene Technologies, Maryland, USA).

### In vitro binding assay

The direct interaction between TRIM50 protein and SNAIL protein were performed by a TNT Quick Coupled Transcription and Translation System (Promega, Madison, WI, USA) according to the manufacturer’s protocol. The TRIM50 and SNAIL proteins were expressed, mixed together, and analyzed with immunoprecipitation by the TRIM50 antibody, followed by western blot assay by SNAIL antibody to determine the direct binding of TRIM50 and SNAIL proteins.

### Subcellular fractionation

Extraction and isolation of nuclear and cytoplasmic protein from HCC cells were performed by the Nuclear and Cytoplasmic Protein Extraction Kit (Beyotime, Jiangsu, China) according to the manufacturer's protocol.

### In vivo tumor growth assay

Five-week-old immunodeficient male BALB/c athymic nude mice (Huafukang Biotechnology Ltd, Beijing, China) were used for construction of xenograft tumor model as described before^15,26^. When visible tumors appeared, we injected 30 ug of pCMV-TRIM50 and empty pCMV vector control to the tumors in either flank once every other day before the mice were sacrificed by cervical dislocation. The isolated tumors in the TRIM50 transfected group and mock control group were further isolated and analyzed.

### Statistical analysis

Statistical analysis was analyzed by SPSS 16.0 software (SPSS, IL, USA) and GraphPad Prism software (version 5.0). Χ2-test was employed to compare qualitative variables. Analysis of quantitative variables was performed using the Student’s *t-*test or one-way analysis of variance (ANOVA). Data were presented as mean ± S.D. *P*-value < 0.05 was considered statistically significant for all tests and all statistical tests were two sided.

## Electronic supplementary material

Supplemental data
