# Correction to: TRIM50 suppressed hepatocarcinoma progression through directly targeting SNAIL for ubiquitous degradation

**DOI:** 10.1038/s41419-018-0835-z

**Published:** 2018-10-09

**Authors:** Xiaoxiao Ma, Xiaomin Ma, Yumin Qiu, Lihui Zhu, Yueke Lin, Yajing You, Dapeng Ma, Zhenzhi Qin, Caiyu Sun, Yunxue Zhao, Yanlin Sun, Lihui Han

**Affiliations:** 1 Department of Immunology, Shandong Provincial Key Laboratory of Infection & Immunology, Shandong University School of Basic Medical Sciences, 250012 Jinan, China; 2 Department of Pharmacology, Shandong University School of Basic Medical Sciences, 250012 Jinan, China; 3 Department of Pathology, Shandong University School of Basic Medical Sciences, 250012 Jinan, China

**Correction to**: *Cell Death Dis.* **9**, 608 (2018); 10.1038/s41419-018-0644-4; Published Online 22 May 2018.

1. In Table 1, the number of patients with distant metastasis should be 13(16.5%), and the number of patients without distant metastasis should be 12(15.4%). The authors placed these two numbers inversely in the published paper.
2. In Fig. 6H, the IHC data is correct. However, a mistake was made in the quantification data of N-cadherin and SNAIL. Expression levels of both of these two proteins are lower in TRIM50 transfected group compared with the mock group. This was caused by a labelling error during the process of quantification analysis. The last two columns of Fig. 6H should be replaced as follows:

**Fig. 6:**
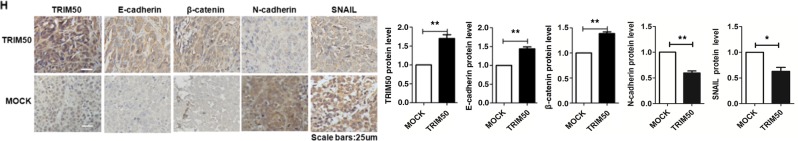
▓

Finally Fig. 6H should be replaced with the following:

The authors apologise for any inconvenience caused by these issues.

