# Supplementary material for: TRIM50 suppressed hepatocarcinoma progression through directly targeting SNAIL for ubiquitous degradation
Source: Cell Death Dis. 2018 May 22;9(6):608. doi: 10.1038/s41419-018-0644-4 (PMC5964248; doi:10.1038/s41419-018-0644-4)

**TRIM50 suppressed hepatocarcinoma progression through directly targeting SNAIL for ubiquitous degradation**

Xiaoxiao Ma^1^, Xiaomin Ma^1^, Yumin Qiu^1^, Lihui Zhu^1^, Yueke Lin^1^, Yajing You^1^, Dapeng Ma^1^, Zhenzhi Qin^1^, Caiyu Sun^1^, Yunxue Zhao^2^, Yanlin Sun^3^, Lihui Han^1^

1. Shandong Provincial Key Laboratory of Infection & Immunology, Department of Immunology, Shandong University School of Basic Medical Sciences, Jinan 250012, China.
2. Department of Pharmacology, Shandong University School of Basic Medical Sciences, Jinan 250012, China.
3. Department of Pathology, Shandong University School of Basic Medical Sciences, Jinan 250012, China.

**Corresponding author**: Lihui Han, M.D., Ph.D., Shandong Provincial Key Laboratory of Infection & Immunology, Department of Immunology, Shandong University School of Basic Medical Sciences, Jinan 250012, China. Phone: 86-531-88382038. Fax: 86-531-8832038. E-Mail: [hanlihui@sdu.edu.cn](mailto:hanlihui@sdu.edu.cn)

**Running Title**：TRIM50 suppressed hepatocarcinoma progression

**Supplementary Tables**

**Supplementary Table 1. Expression of TRIM50 in HCC tissues and non-cancerous liver tissues**

|  | **Cancer(%)** | **Non-cancer(%)** | **χ^2** | **P-value** |
| --- | --- | --- | --- | --- |
| **TRIM50**  **Low**  **High** | **72.1(57/79)**  **27.8(22/79)** | **15.2(12/79)**  **84.8(67/79)** | **48.795** | **0.000***** |

**Note: TRIM50 Low = “–” “+”; High = “++” “+++”.**

**Supplementary Figure Legends**

**Supplementary Figure 1.** TRIM50 reversed resistance to anoikis of HCC cells. BEL7402 cells and HUH7 cells were transfected with TRIM50 plasmid or mock control, followed by anchorage deprival. The anchored and anchorage-deprived cells were presented as attached cells and detached cells, respectively. **(A)** Cell viabilities of these anchorage deprived TRIM50 transfected cells were detected at 0h, 24h, 36h and 48h by CCK8 assay. **(B-C)** HCC cells were transfected with TRIM50 plasmid before anchorage deprival for 24h, and the expression level of cleaved caspase3 was detected by western blot. **(D)** BEL7402 and HUH7 cells were transfected with TRIM50 plasmid or mock control before anchorage deprival for 24h. The detached HCC cells were transferred to six-well plates at the density of 1000 cells per well and allowed to grow for 14 days for colony formation assay. **(E)** Transwell invasive assay were performed to detect the invasive capability of in the detached TRIM50-transfected HCC cells. **P* < 0.05, **P < 0.01 and ***P < 0.001 for statistical analysis of the indicated groups.

**Supplementary Figure 2.** The expression of TRIM50 and SNAIL in clinical HCC patients and corresponding non-cancerous liver tissues was detected by IHC. Expression levels of TRIM50 in patients with lower expression of SNAIL and patients with higher expression level of SNAIL were statistically analyzed. Typical images were presented (left panel) and the expression levels of TRIM50 were statistically analyzed and compared(right panel). **P* < 0.05 for statistical analysis of the indicated groups.

**Supplementary Figure 3.** BEL7402 cells were transfected wit TRIM50 expression plasmid or mock control and further cultured for 24h. The photographs of these transfected cells were taken and typical images were presented.

**
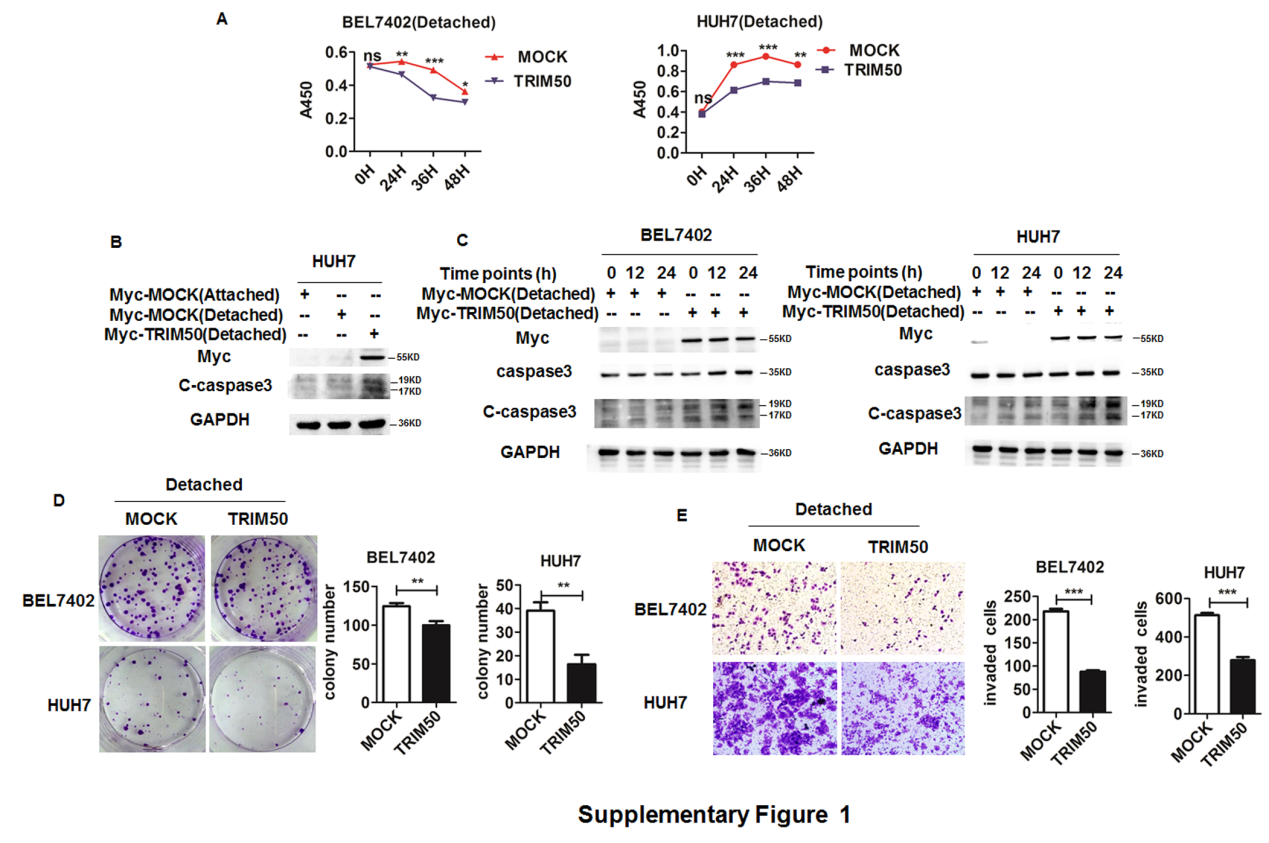
**


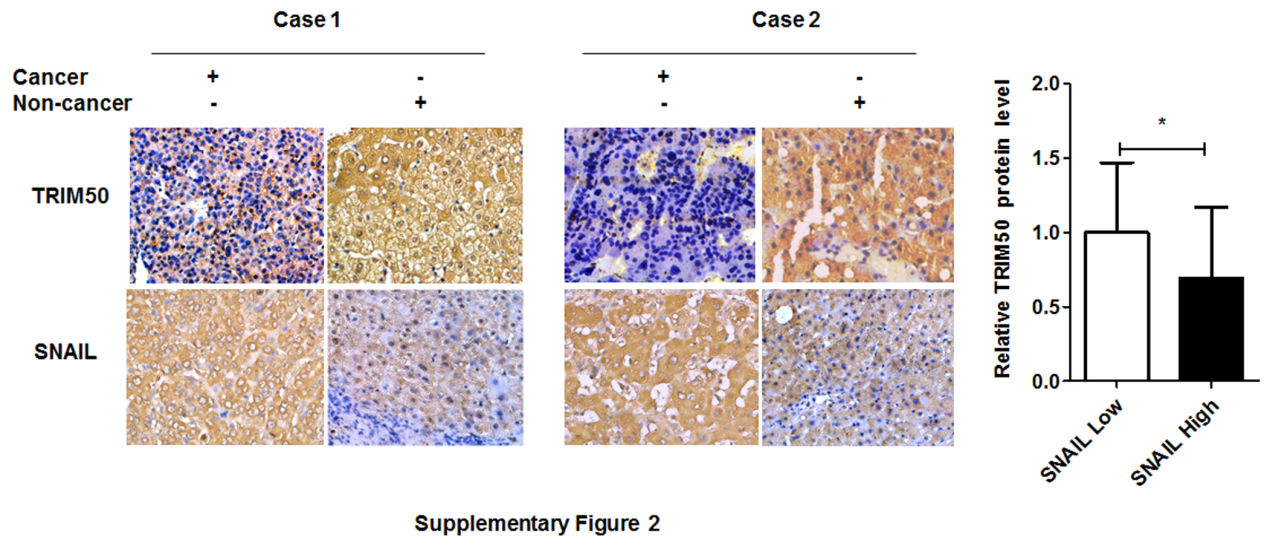


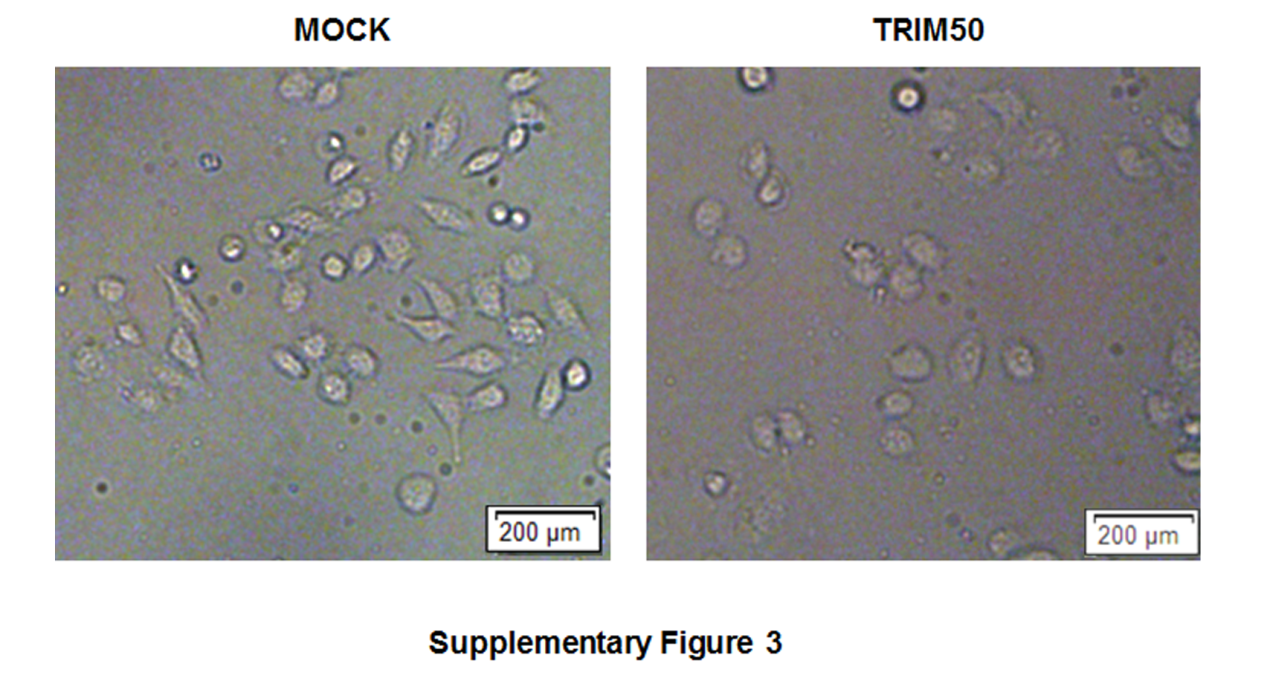

Supplement: Supplementary file 1 — Supplemental data [file 41419_2018_644_MOESM1_ESM.docx]
